# Relations between subjective well-being and Alzheimer’s disease: A systematic review

**DOI:** 10.1590/1980-57642020dn14-020008

**Published:** 2020

**Authors:** Fernanda Panage Moura, Amer Cavalheiro Hamdan

**Affiliations:** 1 Psychologist and student of the Postgraduate program in Psychology of UFPR, Curitiba, PR, Brazil.; 2 Professor Doctor of the Postgraduate Program in Psychology of UFPR, Curitiba, PR, Brazil.

**Keywords:** Alzheimer disease, subjective well-being, aged, health, doença de Alzheimer, bem-estar subjetivo, idosos, saúde

## Abstract

Subjective Well-Being (SWB) is determined by the degree of satisfaction with one's own life and the intensity/frequency with which we experience negative and positive emotions. Current studies indicate that SWB is beneficial for health.

**Objective:**

The aim of this systematic review was to analyze the methodological quality of published articles on SWB in people with Alzheimer’s disease (AD).

**Methods:**

The keywords “Well-Being” and “Alzheimer” were used. Inclusion criteria were a) articles with a sample of the elderly population; b) empirical articles; c) articles published between 2014 and 2019. Analysis of the selected articles was performed using the Downs and Black Checklist.

**Results:**

13 articles were selected for further analysis. The results showed that only one of the articles reached a high methodological quality level. The other articles had an average level, ranging from 46% to 67%, of total protocol compliance.

**Conclusion:**

The studies analyzed had a medium level of methodological quality. It is important to improve the methodological quality of studies on SWB in people with AD.

## RESUMO.

O bem-estar subjetivo (SWB) é determinado pelo grau de satisfação com a própria vida e pela intensidade/frequência que experimenta emoções negativas e positivas. Atualmente, existem estudos indicando que o SWB é benéfico para a saúde. **Objetivo:** O objetivo desta revisão sistemática foi analisar a qualidade metodológica dos artigos publicados sobre aspectos do bem-estar subjetivo em pessoas com doença de Alzheimer.

**Métodos:**

Foram utilizadas as palavras-chave “Bem-Estar” e “Alzheimer”. Os critérios de inclusão foram: a) artigos com amostra da população idosa; b) artigos empíricos; c) artigos publicados de 2014 a 2019. A análise dos artigos selecionados foi realizada por meio da “Checklist Downs and Black”.

**Resultados:**

13 artigos foram selecionados para análise. Os resultados indicam que apenas um dos artigos atingiu um alto nível de qualidade metodológica, conforme protocolo utilizado. Os demais atingiram um nível médio, variando de 46% a 67% da total adesão ao protocolo.

**Conclusão:**

Os estudos analisados ​​apresentam um nível médio de qualidade metodológica. É importante melhorar o método de estudos que visam estudar os aspectos do bem-estar subjetivo em pessoas com DA.

Global population aging is progressing at an increasingly rapid pace. The accelerated growth of the elderly population has important implications for social, economic and environmental factors, and calls for a comprehensive public health response.^1^ However, according to the World Health Organization,^1^ these issues have not been sufficiently debated, and few solutions to these problems have been proposed. Thus, there is a clear need to intensify the study of health among the elderly. According to the World Health Organization^2^ 47.5 million people are estimated to be living with dementia (1, the coming years will see a significant increase in the incidence of dementia in developing countries. In 2010, the worldwide prevalence of dementia was approximately 35.6 million, with Alzheimer’s disease (AD) alone accounting for 25 million cases.

AD is a chronic neurodegenerative disease that leads to impairments in memory, language, problem-solving and other cognitive skills, including executive functions. Alterations in cognitive functions affect the ability to perform daily activities and often interfere with motivation, emotional control, and social behavior. As the illness progresses, patients become partly to fully dependent on family members or other caregivers. This can compromise the health of the patient and of the caregivers involved, as well as affecting their quality of life and well-being.^3^

Subjective well-being (SWB), considered by some as synonymous with quality of life, is a widely studied concept in the field of positive psychology. SWB is composed of reflexive cognitive judgments, such as life satisfaction, and emotional responses throughout life, such as pleasant and unpleasant emotions. Thus, SWB is determined by the degree of satisfaction with one’s life and the intensity and frequency with which the individual experiences positive and negative emotions.^4^

In recent years, several studies have sought to investigate the possible repercussions of high levels of SWB. These investigations have revealed significant benefits of SWB on health, longevity, supportive social relationships, job performance and resilience.^4^^,^ 5 Studies such as those of Sadler^6^ and Sargent-Cox^7^ have also identified associations between life satisfaction and increased longevity, the adoption of healthy behaviors and an improved immune response.

The study of SWB necessarily relies on self-assessment, since life satisfaction is individually determined. However, self-assessments can be especially challenging for patients with AD, who may have poor insight, reduced ability to recognize changes or difficulty choosing among multiple alternatives. Nevertheless, recent studies suggest that patients with mild to moderate dementia can still provide a reliable assessment of their levels of well-being.^6^^,^ 7

In light of this evidence and global trends in the incidence of AD, there is a growing need to intensify and refine the study of SWB in this population. Therefore, the aim of this systematic review was to analyze the methodological quality of published articles on aspects of SWB in AD. The findings of this review may help future studies achieve better methodological quality and obtain more consistent results.

## METHODS

This study was conducted according to the Preferred Reporting Items for Systematic Reviews and Meta-Analyses (PRISMA) checklist.^8^ The main goal of the present review was to assess the methodological quality of studies of well-being in patients with AD.

The articles were retrieved and screened by both authors. In June 2019, electronic searches were conducted on the following databases: Scopus, PsycInfo, and PubMed. The keywords “Well-Being” and “Alzheimer” were used. Inclusion criteria were as follows: a) articles involving samples of elderly participants; b) empirical studies; and c) articles published between 2014 and 2019. After the exclusion of duplicate articles (56), a total of 186 articles were identified.

**Figure 1:**
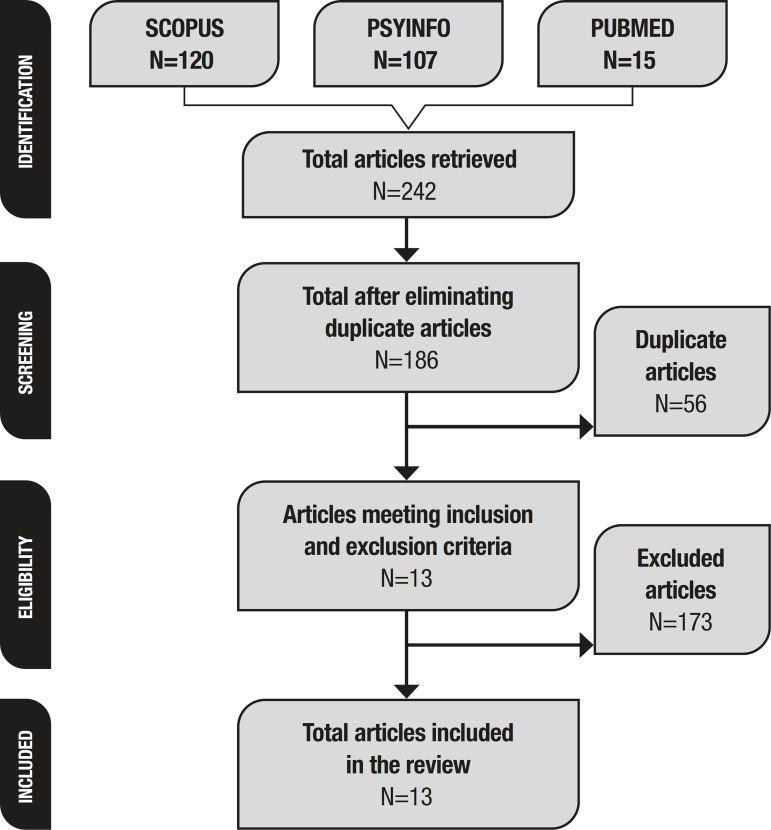
Article screening and selection.

The abstracts of the articles were first screened for inclusion and exclusion criteria. Figure 1 presents a summary of the screening and selection process. The following exclusion criteria were applied: a) Studies of participants without AD (43 articles excluded); b) articles involving caregivers only (53 articles excluded); c) articles that did not evaluate well-being (49 articles excluded); d) psychometric studies (28 articles excluded). The following data were extracted from each article: 1) Authors; 2) Design; 3) Sample and 4) Conclusion. This data can be found in Table 1.

**Table 1:** Data extracted from selected articles (n=13).

Author (year)	Design	Sample (IG; CG)	Outcomes
Koivisto et al. (2016)^10^	Randomized longitudinal study	236 (152; 84)	Psychosocial intervention had no effect on well-being, disease progression, or AD symptoms.
Silva et al. (2017)^11^	Randomized longitudinal study	51 (34; 17)	Memory rehabilitation training had positive effects on the well-being of patients with AD (in the short term).
Narme et al. (2014)^12^	Randomized longitudinal study	48 (24; 24)*	Musical interventions may improve the well-being of patients with AD.
El-Kader and Al-Jiffri (2016)^13^	Randomized longitudinal study	59 (29; 30)	Treadmill training is effective for improving QOL, systemic inflammation and psychological well-being in people with AD.
Woods et al. (2014)^14^	Cross-sectional study	101	Level of awareness of deficits has little influence on QOL assessments in dementia.
Todri et al. (2019)^15^	Randomized longitudinal study	174 (100; 74)	Controlled and supervised GPR postural technique is valid to improve well-being but lacks evidence of effectiveness.
Orgeta et al. (2015)^16^	Cross-sectional study	488	Self-rated health in people with AD and their caregivers provides important information regarding determinants of QOL in dementia.
Daley et al. (2017)^17^	Cross-sectional study	58	Preserved emotional perception skills in participants with AD are not related to satisfaction with relationships to caregivers.
Wettstein et al. (2014)^18^	Cross-sectional study	257	OOHB was effective for improving activities of daily living and QOL of individuals with AD.
Cines et al. (2015)^19^	Cross-sectional study	104	Preserved cognitive skills improve psychological well-being in AD.
Ismail et al. (2018)^20^	Randomized longitudinal study	29 (13; 16)*	The nostalgia intervention boosted self-reported psychological resources, positive affect and meaning in life.
Larouche et al. (2019)^21^	Randomized longitudinal study	48 (24; 24)*	Mindfulness and MAT Intervention have the potential to reduce depressive and anxious symptoms and improve QOL in AD.
Stites et al. (2018)^22^	Cross-sectional study	259 (160; 99)	Cognitive complaints are associated with low QOL, higher depression, anxiety, stress and poor well-being.

IG: intervention group; CG: control group;

* both intervention groups; QOL: quality of life; GPR: Global Postural Reeducation; OOHB: out-of-home behavior; MAT: Monitoring and Acceptance Theory.

The selected articles were analyzed using the Downs and Black Checklist^9^ internal validity (bias and confounding. This instrument was developed for the methodological evaluation of randomized and observational studies. The checklist yields a maximum score of 28 based on 27 items divided into 5 evaluation domains: Report (RD); External Validity (EVD); Bias (BD); Confounding (CD); and Power (PD). Each item receives a rating of “1” (when the criterion is met in the study) or “0” (when the criterion is not met). The exception to this is the RD domain, which can receive a score of “2” (when the criterion is met), “1” (when the criterion is partially met) or “0” (criterion is not met in the study). Higher scores are indicative of better methodological quality. The authors used these scores to classify the studies into one of three groups: low/poor quality - achieving less than 40% of the maximum score; moderate/reasonable quality - achieving 40% to 70% of the maximum score; high/good quality - achieving at least 70% of the maximum score.

## RESULTS

The electronic search retrieved a total of 242 articles. After inclusion and exclusion criteria were applied, 13 articles were selected for this review.

Of the 13 articles included, 62% consisted of longitudinal studies while the remainder were cross-sectional. The assessment of sample sizes showed that 31% of studies involved 50 participants or fewer, and 31% worked with samples of over 200 participants. Most of the studies (46%) were published in 2014 and 2015. Only two of the articles included (15%) had been published in 2019 (Table 1), although this may be because the search was conducted in June of that same year.

Six of the 13 articles described interventions which had positive effects on the SWB of people with AD (Table 1). The interventions included the following: memory rehabilitation; musical interventions; walking training; mindfulness intervention; out-of-home behavior training; and nostalgia intervention.

### Methodological quality analysis

The highest total score obtained by any article on the Downs and Black Checklist was 20 out of a possible 28 points (71% of quality criteria met). The average score of the 13 articles was 16 (58% of quality criteria met), and the lowest total score was 12 (43% of quality criteria met). These results indicate that only one of the articles had high methodological quality, with the majority of studies obtaining average scores and meeting 46% to 67% of the criteria in the checklist.

#### Reporting (RD)

The studies obtained satisfactory scores in the reporting domain, ranging from 7 to 10 out of a maximum of 11 points. This domain evaluates the quality, clarity and objectivity of descriptions of the method and results. All articles provided a satisfactory description of their objectives, main outcomes, sample characteristics and results. They also clearly described the interventions performed. However, only the studies by Ismail et al.^20^ and Woods et al.^14^ provided detailed information about all important adverse events in the study.

#### External validity (EVD)

Overall, the studies showed low external validity, with this domain showing the lowest scores out of all five domains in the checklist. The scores of the articles included in the review ranged from 0 to 1, out of a total of 3 points, with a mean score of 0.17. Most articles did not present information to indicate whether the studied sample was representative of the target population or if the circumstances under which the interventions were implemented (e.g. location and intervention teams) were representative of those available to the population. The only studies which met some of the criteria in this domain were those of Koivisto et al.^10^ and Silva et al.,^11^ which provided information about the extent to which the places where the interventions were performed was representative of the locations available to the studied population.

#### Bias (BD)

The studies obtained satisfactory scores in the bias domain, ranging from 3 to 6 out of a total of 7 points. The average score obtained by the 13 articles was 5. This domain evaluates measurement biases in the intervention and outcomes of the study. Only a few studies, such as that of El-Kader and Al-Jiffri.^13^ sought to ensure the participants were blind to group allocation. However, most studies used adequate statistical tests, showed adherence to intervention protocols and provided reliable and valid measurements of main outcome variables.

#### Confounding (CD)

The articles had a medium risk of confounding bias. Scores on this domain ranged from 1 to 5 out of a maximum of 6 points, with a mean score of 3. This domain refers to any efforts made toward using measures that minimize the effects of confounders, unwanted variables that may interfere with the results of the study. Although most articles relied on randomized allocation and considered sample loss in the intervention, most did not adjust for confounding variables in their analyses.

#### Power (PD)

Power scores were the second lowest of the five domains, suggesting low levels of statistical power across the studies included. Scores in this domain ranged from 0 to 1, out of a maximum of 1 point, with a mean of 0.38. This domain refers to the probability that findings obtained in the study were not attributable to chance and evaluates whether the study has sufficient power to detect a clinically important effect. Only 5 out of 13 studies presented sufficient evidence of statistical power. The studies in question were those of Woods et al.,^14^ Orgeta et al.,^16^ Daley et al.^17^, Wettstein et al.,^18^ and Larouche et al.^21^ These studies provided beta values for their statistical analyses, which helped substantiate the robustness of their results.

### Outcome analysis

Six studies obtained positive findings regarding the SWB of people with AD. The first study, conducted by Silva et al.,^11^ evaluated an intervention which relied on paper and pencil tasks to improve attention, working memory, autobiographical and episodic memory, semantic memory and implicit memory. The exercises had increasing levels of difficulty, and each session involved two explicit memory and one implicit memory exercise.

In the second study, conducted by Narme et al.,^12^ the intervention involved music and cooking activities. During music sessions, music was played on a CD player and the participants were asked to listen and follow along by singing or using percussion instruments. In cooking sessions, participants were asked to make a different recipe at each session, and were encouraged to express any feelings or autobiographical memories evoked by the activity. This intervention had short- and long-term effects on emotional, cognitive, and behavioral outcomes.

In the third study, authored by El-Kader and Al-Jiffri,^13^ the intervention was aerobic exercise on a treadmill. The training program started with a 5-minute warm-up (range of motion and stretching exercises), followed by 10-30 minutes of aerobic exercise training and a 10 minutes cool-down on the treadmill at low speed and no inclination. Participants completed 3 sessions/week for 2 months with close supervision by a physical therapist. The results showed that treadmill walking exercise was effective at improving quality of life and psychological well-being in AD.

The fourth study, by Wettstein et al.,^18^ was cross-sectional, and focused on out-of-home behavior (OOHB), which includes the full range of activities performed outdoors. In this study, in addition to OOHB, the following variables were evaluated: Walking distance, Walking duration, Walking speed, Number of nodes visited, Time out of home, Number of cognitively demanding activities and Number of physically demanding activities. The results suggested that OOHBs are a challenge for elderly individuals with cognitive impairment and are related to aspects of well-being.

The fifth study was authored by Ismail et al.^20^ and investigated the potential of nostalgia to improve psychological well-being among people with dementia. Participants were asked to evoke and describe the past event which made them most nostalgic. In a second experiment, nostalgia was induced using music. Participants were asked to listen to a song and describe any past event or experience associated with it. Results suggested that nostalgia boosted self-reported psychological resources, specifically positive affect and meaning in life.

Finally, the sixth study, by Larouche et al.,^21^ evaluated the benefits of a mindfulness-based intervention (MBI). The program comprised eight sessions lasting two and a half hours each. Every session included a guided meditation exercise, group discussions about meditation and home practices, and psychoeducation about mindfulness, stress management and overcoming obstacles. Results confirmed the potential of MBI to reduce depressive and anxious symptoms in older adults with AD.

## DISCUSSION

This review aimed to analyze the methodological quality of studies of SWB in people with AD. The results showed that studies had average methodological quality. According to the evaluation performed, the article with the best methodological quality was that of Larouche et al.,^21^ followed by the studies of Koivisto et al.,^10^ Silva et al.,^11^ Todri et al.^15^ this study has shown interest in evaluating the effects of Global Postural Reeducation (GPR and Ismail et al.^20^ The lowest scores were obtained by the studies of Stites et al.^22^ and Orgeta et al.,^16^ both of which had a cross-sectional design.

Among the five domains evaluated, the lowest scores were observed in the EVD and PD domains, where most studies were classified as having low methodological quality. These domains refer to the consistency of the results, as well as their validity and generalizability. Studies that do not meet these criteria may present inconsistent results and conclusions, revealing the need for improvements in methodological quality and further studies of SWB in AD.

Most studies recruited participants, selected the sample and performed interventions in a single health care center for patients with AD. The descriptions of participant recruitment and selection were unclear and lacking in detail, making it difficult to determine whether their samples were representative of the target population. These were the most frequent reasons for the low scores obtained by most studies in the EVD domain. The poor methodological quality in the PD domain was mostly attributable to statistical flaws, including missing information and the absence of beta (b) coefficients.

The best scores on the checklist were obtained in the RD and BD domains, where most studies were classified as having reasonable-to-good methodological quality. In the CD domain, the studies obtained average scores, suggesting reasonable quality. These findings suggest that the articles were clearly written and well-structured, with a reasonable level of consideration and control of confounding variables.

Some aspects of this review may be considered methodological limitations, such as the use of only 3 databases for the search and selection of studies. The restrictions placed on the publication period may have led to the exclusion of some studies of SWB in people with AD. Another potential limitation is the quality assessment method itself: the Downs and Black Checklist may not include all relevant aspects of the research method, so it should be used with caution.^23^

In conclusion, the studies analyzed had average methodological quality, which should be considered a reasonable classification. Nevertheless, there is a need to improve and enhance the methodological characteristics of studies of SWB in people with AD, paying special attention to external validity and the generalizability of results. To this end, it is crucial that future studies provide more detailed and comprehensive descriptions of statistical analyses and corresponding results.
